# Haemodynamic determinants of supine hypertension in patients with classical orthostatic hypotension

**DOI:** 10.1097/HJH.0000000000004194

**Published:** 2025-11-13

**Authors:** Amber H. van der Stam, Boriana S. Gagaouzova, Fabian I. Kerkhof, Ineke A. van Rossum, Sharon Shmuely, Robert H. Reijntjes, Marc J. van Houwelingen, Roland D. Thijs, J. Gert van Dijk

**Affiliations:** a Radboud University Medical Center, Donders Institute for Brain, Cognition and Behavior, Department of Neurology, Center of Expertise for Parkinson & Movement Disorders, Nijmegen; b Department of Neurology, Leiden University Medical Centre, Leiden; c Stichting Epilepsie Instellingen Nederland (SEIN), Heemstede; d Department of Experimental Cardiology, Erasmus Medical Centre, Rotterdam, the Netherlands; e UCL Queen Square Institute of Neurology, University College London, London, UK

**Keywords:** autonomic dysfunction, tilt table test, total peripheral resistance

## Abstract

**Objective:**

The relation between classical orthostatic hypotension (cOH) and supine hypertension is largely unknown. We investigated the relative contributions of heart rate (HR), stroke volume (SV) and total peripheral resistance (TPR) to supine and upright blood pressure (BP).

**Methods:**

In this retrospective study, tilt tests were divided in four groups: 19 normotensive and 61 hypertensive controls, 50 cOH patients with SH (cOH/SH+) and 30 without (cOH/SH−). Hypertension was defined as supine SBP at least 140 mmHg. We used linear regression to relate cOH severity to supine SBP, and the logratio method to analyse relative contributions of HR, SV and TPR. *P* values less than 0.003 were considered significant.

**Results:**

High supine SBP was associated with high TPR in patients and controls. Orthostatic SBP decrease in cOH was larger in those with higher supine SBP. The main parameter explaining this effect was a high supine TPR that did not increase after tilt in cOH/SH+ compared to cOH/SH− (logratio difference, *P* < 0.002). SV logratio decreased more in cOH/SH− than in cOH/SH+ (*P* < 0.003), and HR logratio contributed similarly to orthostatic SBP in both cOH groups (*P* = 0.028).

**Conclusion:**

While high supine TPR explained SH, a failure to further increase upright TPR explained the orthostatic SBP fall in patients. Autonomic failure can explain the SBP fall but not directly the high supine TPR that causes SH. We assume that slow-acting humoral vasoconstrictors are triggered in the upright position and continue to act after tilting back, causing high TPR and SH.

## INTRODUCTION

Orthostatic hypotension is common and associated with complaints in the upright position such as dizziness and syncope. Classical orthostatic hypotension (cOH) is defined as a drop in SBP of at least 20 mmHg or a DBP drop of at least 10 mmHg within 3 min of standing or after tilt during a tilt table test (TTT) [1]. There are many possible causes of cOH, such as hypovolemia, medication use or heart failure, as well as disorders of autonomic nervous control that result in baroreflex failure [2,3]. When the latter regards efferent nerve fibres, the result is called neurogenic orthostatic hypotension (nOH), occurring, for example, in multiple system atrophy, Parkinson's disease and pure autonomic failure [4].

A problem frequently associated with nOH is supine hypertension (SH). SH may occur in 50% of a nOH population [5,6]. Both cOH and SH impact quality of life and carry risks of damage to kidneys, heart and brain. cOH and SH are independently related to cardiovascular and noncardiovascular mortality, making them important treatment targets [7–12].

The mechanisms of SH are unclear [6]. Some authors sought explanations for SH in neurohumoral effects, such as a reduction in plasma noradrenalin in those with peripheral neurodegeneration [13], or in residual sympathetic tone in central autonomic dysfunction like multiple system atrophy [6,14,15]. Various consensus statements described an association between SH and the severity of orthostatic hypertension, defined here as the magnitude of the blood pressure (BP) decrease after assuming the upright position [1,16]. The mechanism of this association is uncertain.

The three haemodynamic components of mean arterial pressure (MAP) are total peripheral resistance (TPR), stroke volume (SV) and heart rate (HR). We recently found that a failure of TPR to increase in the upright position was the main mechanism behind cOH [13,14,17].

Here, we compare haemodynamic parameters of SH in cOH patients to those causing hypertension in controls, determine whether the severity of cOH is related to SH, and explore the mechanism of this association. The logratio method allows a fully quantitative comparison of relative BP changes using HR, SV and TPR [18,19]. As the contributions of HR, SV and TPR in various causes of cOH are largely unknown, we studied cOH regardless of cause and did not a priori distinguish between nOH and non-nOH [17].

## METHODS

### Population

**FIGURE 1:**
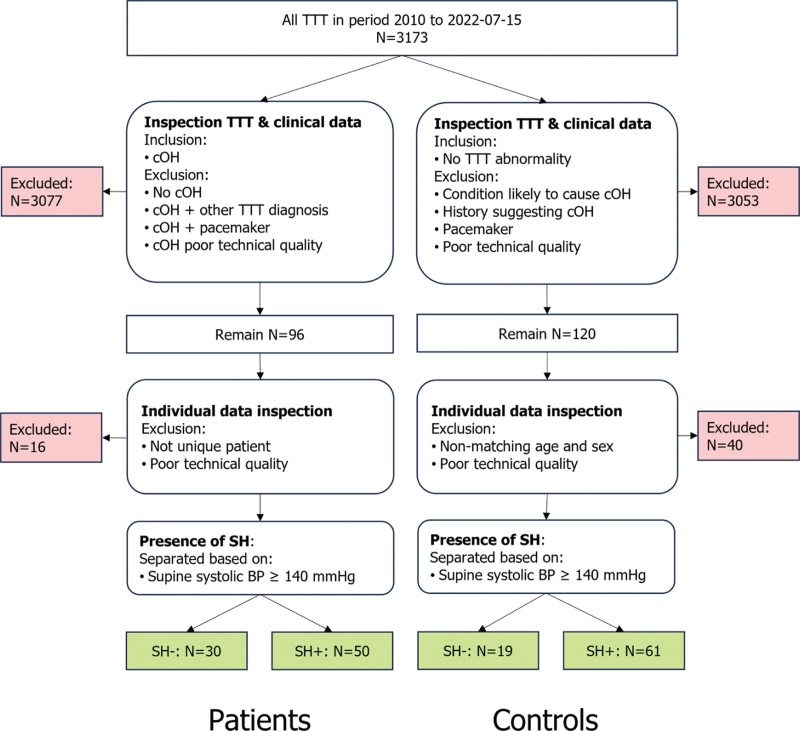
Flow diagram of participant selection. Evaluation of the tilt table test recordings and reports when selecting the patients and controls to include in the analysis.^17^ BP, blood pressure; cOH, classical orthostatic hypotension; SH, supine hypertension; TTT, tilt table test.

The study was retrospective and based on the TTT database of Leiden University Medical Centre (LUMC, Leiden, The Netherlands). All patients were seen at the department of Neurology between January 2010 and July 2022 as part of regular care after being referred for syncope or suspected autonomic problems. In this period, 3173 TTTs were performed (Fig. 1). TTT records were first selected based on technical quality. To qualify as cOH, current definitions of cOH had to be met (i.e. a sustained SBP decrease of at least 20 mmHg or DBP decrease of at least 10 mmHg within 3 min of tilt). We included all cases of cOH, not confining data to any circumscribed group. The results may, therefore, represent various underlying conditions, although patients with known causes of neurogenic cOH formed the majority [17]. Patients were excluded if they had a pacemaker or additional TTT abnormalities. The control population comprised people who visited the outpatient clinic for dizziness but who exhibited no abnormalities during TTT, did not have autonomic dysfunction, and in whom complaints were likely due to a noncirculatory origin. Controls were groupwise matched for sex and age. Medication use and reason for the hospital visit were noted. We stress that in this patient group, medication use did not affect the haemodynamic pattern of cOH [17]. As the test was exclusively performed as part of regular care, medical–ethical approval was not needed according to Dutch law.

### Tit table test protocol

The LUMC TTT protocol consisted of a rest period of 10 min followed by 70° head-up tilt for 20 min or until syncope or limiting complaints of presyncope occurred. Continuous BP measurements were gathered with the noninvasive volume clamp method using either the Finapres NOVA (Finapres Medical Systems, Enschede, The Netherlands) or the Nexfin (BMEye, Amsterdam, The Netherlands). At least one electrocardiography lead and video-electroencephalography were part of the standard procedure [20,21].

### Haemodynamic analysis

Video records were reviewed to extract the time of head-up tilt and other events with an accuracy of 1 s. Modelflow was used to derive beat-to-beat data of MAP, SBP, DBP, HR, SV and TPR. Modelflow is a physiological model that uses the shape of the continuously recorded SBP curve per heart beat to infer stroke volume; together with MAP and HR, this allows calculation of TPR [19,22]. All six haemodynamic variables were resampled at 1 Hz using linear interpolation and interactively cleaned of artifacts caused by extrasystolic beats, movement or technical issues, using purpose-written software. Periods with artifacts were interpolated if they lasted less than 5 s and were flanked by stable data; if not, they were deleted.

Periods up to 6 min before tilt and up to 10 min after tilt were extracted. The beginning and end of this period could be shortened to avoid contamination with a different condition, e.g., active stand, another test or a change in posture. To illustrate the temporal course of events, continuous haemodynamic data were plotted, but to simplify the quantitative analysis we reduced measurements to only the baseline and upright positions. As a measure of baseline supine values, we averaged measurements per person over 160 s, lasting from 180 s to 20 s before tilt for all variables (MAP, SBP, DBP, HR, SV and TPR). As a measure of the orthostatic condition, we averaged data over 60 s, from 180 to 240 s after tilt (i.e. the fourth minute), corresponding with the convention to assess cOH 3 min after assuming the upright position.

Patient and control groups were each split into two groups, based on the consensus for SH in patients with nOH: a SBP of at least 140 mmHg or DBP of at least 90 mmHg, after at least 5 min rest in the supine position [16]. The groups were labelled SH+ and SH−, recognizing that the term ‘SH’ is strictly speaking not intended for use in controls without cOH. For a post hoc analysis of the relationship between the DBP and the reduction in SBP upon tilt, all patients were split into a low DBP group and a high DBP group according to the median of the supine DBP.

### Logratio analysis

First, we calculated the average of the supine baseline period for each person for MAP, HR, SV and TPR. The values for the entire time series were divided by this average value, resulting in time series of ratios with a value of 1 for the baseline period. The logarithm was then taken of all these values, resulting in time series of MAP_LR_, HR_LR_, SV_LR_ and TPR_LR_. Note that MAP_LR_ is the sum of HR_LR_, SV_LR_ and TPR_LR_ for each point in time. A negative logratio indicates a reduction compared to the baseline supine value, and a positive logratio indicates an increase [19,23]. For the statistical analysis, the average of upright values were calculated per person over the fourth minute after tilt. All analyses were performed in Matlab version R2022a.

### Statistics

We compared haemodynamic parameters of the fourth minute after tilt with supine values. As not all data were normally distributed, we used the two-tailed Mann-Whitney *U* test throughout for consistency. To analyse the BP change in response to upright tilt, we applied Pearson's linear regression, comparing the difference in SBP between the upright and supine position to the supine value. To correct for multiple testing, the Bonferroni correction was applied for 18 comparisons (Table 2), resulting in a significance threshold of *P* < 0.003. BP comparisons between SH+ and SH− groups were excluded from this correction, because these differed by definition. We reported a result as a trend when the *P* value was 0.003 < *P* < 0.01. For post hoc analyses of interindividual variability in haemodynamic control, Fisher's exact test was used, with a significance threshold of *P* < 0.05.

**TABLE 1:** Demographics of the population

Demographics						
	Control total (*n* = 80)	Control SH− (*n* = 19)	Control SH+ (*n* = 61)	cOH total (*n* = 80)	cOH SH− (*n* = 30)	cOH SH+ (*n* = 50)
Age [years; median (range)]	65 (50–87)	61 (50–75)	67 (50–87)	68 (43–90)	65.5 (43–79)	68.8 (50–90)
Female (%)	26 (33%)	4 (21%)	22 (36%)	24 (30%)	7 (23%)	17 (34%)
Duration cOH [months; median (range)]	–	–	–	24 (1–288)	36.1 (3–120)	42.6 (1–288)
Diagnosis						
PAF	–	–	–	8	3	5
MSA	–	–	–	10	4	6
PD	–	–	–	27	10	17
Other nOH (likely)	–	–	–	13	4	9
Diabetes mellitus	–	–	–	3	0	3
Drug induced OH	–	–	–	4	1	3
Other non-nOH	–	–	–	15	8	7
Medication						
BP-increasing drugs	2	0	2	19^a^	8	11^a^
BP-lowering drugs	42	6	36	50	17	30

Clinical profiles of all controls and patients with classical orthostatic hypotension (cOH) are displayed as well as the subgroups separated based on the presence (+) or absence (−) of supine hypertension (SH). BP, blood pressure; cOH, classical orthostatic hypotension; MSA, multiple system atrophy; nOH, neurogenic orthostatic hypotension; PAF, pure autonomic failure; PD, Parkinson's disease; SH, supine hypertension.

a Two patients received a combination therapy of midodrine and fludrocortisone.

**TABLE 2:** Haemodynamic parameters

	Control SH− (*n* = 19) Median (IQR)	Control SH+ (*n* = 61) Median (IQR)	*P* value	cOH/SH− (*n* = 30) Median (IQR)	cOH/SH+ (*n* = 50) Median (IQR)	*P* value
Supine						
MAP (mmHg)	92.9 (82.8–101.1)	115.8 (107.3–122.9)	8.1 × 10^–9^	89.5 (80.6 - 94.3)	121.6 (109.7 - 137.5)	7.0 × 10^−13^
SBP (mmHg)	124.6 (114.4–130.3)	159.0 (148.3–171.1)	5.9 × 10^−11^	125.6 (111.8–134.4)	169.8 (159.1–189.6)	9.4 × 10^−14^
DBP (mmHg)	72.9 (65.3–80.0)	88.2 (80.7–93.6)	3.4 × 10^–6^	66.5 (61.9–73.4)	86.7 (79.4–102.0)	8.3 × 10^−11^
HR (bpm)	67.0 (61.7–76.0)	66.2 (62.4–78.8)	0.769	67.1 (61.9–73.4)	70.4 (64.4–76.2)	0.212
SV (ml)	67.8 (56.0–89.6)	63.9 (52.4–77.4)	0.288	75.7 (64.2–87.4)	73.1 (56.5–83.1)	0.151
TPR (mmHg s/ml)	1.0 (0.8–1.6)	1.6 (1.1–2.0)	0.0067	1.0 (0.8–1.2)	1.4 (1.2–2.0)	1.5 × 10^−5^
Fourth minute 70° tilt						
MAP	98.2 (86.9–112.9)	124.6 (114.3–131.6)	3.6 × 10^–7^	73.4 (67.7–79.6)	96.2 (82.4–104.8)	1.4 × 10^−6^
SBP	123.0 (114.9–140.7)	163.5 (152.8–180.7)	3.6 × 10^–9^	95.1 (84.2–103.1)	124.8 (103.5–141.9)	2.0 × 10^−7^
DBP	81.2 (69.0–93.8)	95.9 (88.9–107.4)	2.1 × 10^–4^	63.3 (54.7–67.4)	76.9 (64.0–90.4)	2.2 × 10^−5^
HR	76.8 (70.1–82.8)	72.5 (64.9–82.9)	0.314	80.6 (72.5–88.8)	77.8 (71.9–87.0)	0.709
SV	51.7 (40.1–72.5)	47.7 (39.2–66.1)	0.498	47.2 (40.1–55.3)	52.2 (35.8–58.9)	0.673
TPR	1.4 (1.1–2.1)	2.1 (1.5–2.7)	0.012	1.1 (0.8–1.6)	1.3 (1.1–2.1)	0.016
Logratio fourth minute 70° tilt						
MAP_LR_	0.032 (0.008–0.046)	0.027 (0.002–0.055)	0.643	−0.064 (−0.116 to −0.046)	−0.090 (−0.161 to −0.055)	0.047
HR_LR_	0.046 (0.025–0.064)	0.021 (0.009–0.047)	0.018	0.058 (0.045–0.107)	0.045 (0.018–0.081)	0.028
SV_LR_	−0.125 (−0.174 to −0.083)	−0.119 (−0.151 to −0.069)	0.325	−0.196 (−0.226 to −0.174)	−0.150 (−0.197 to −0.108)	1.5 × 10^−3^
TPR_LR_	0.110 (0.069–0.153)	0.105 (0.054–0.175)	0.709	0.055 (0.016–0.085)	−0.002 (−0.055 to 0.054)	0.0025

Comparisons of the three parameters and blood pressure during head-up tilt testing. The comparisons with a *P* value smaller than 0.01 were classified as a trend, and due to Bonferroni correction those with a *P* value below 0.003 classified as significant. cOH, classical orthostatic hypotension; HR, heart rate; IQR, interquartile range; LR, logratio; MAP, mean arterial pressure; NS, not significant; SH, supine hypertension; SV, stroke volume; TPR, total peripheral resistance.

## RESULTS

We selected 160 TTT records: 80 patients and 80 controls (Table 1). Fifty cOH patients (62.5%) and 61 controls (76.3%) met the systolic SH criterion and were categorized as SH+; the diastolic criterion for SH was met by 24 cOH patients (30%) and 25 controls (31.3%), all of whom already met the systolic criterion. Controls without SH were younger than controls with SH (*P* = 0.035, Table 1). For patients with cOH, there was no difference in age between the two subgroups.

### Haemodynamic variables

**FIGURE 2:**
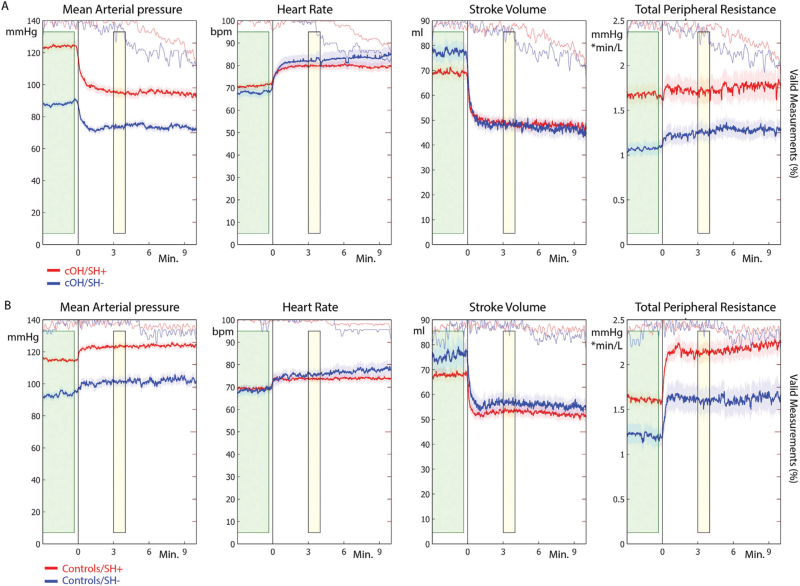
Haemodynamic changes over time. For (a) patients with classical orthostatic hypotension (cOH) (*n* = 80) and (b) controls (*n* = 80), the mean arterial pressure (MAP), heart rate (HR), stroke volume (SV) and total peripheral resistance (TPR) are plotted as a group average with one standard error. Both cOH patients and controls were divided using a threshold of supine SBP of 140 mmHg, resulting in a supine hypertension group (SH+, red) and a supine normotension group (SH−, blue). Thin pale-coloured lines indicate the percentage of measurements valid for each group at each point in time, with 100% at the top of the right-hand axis. The vertical line at 0 s indicates the moment the act of tilting up was completed. The green rectangle indicates the period used for the supine baseline, and the yellow rectangle highlights the fourth minute of the measurement used to quantify the upright position.

SH+ groups for both controls and cOH patients had, by definition, higher supine MAP, SBP and DBP than SH− groups, which persisted after tilt (Fig. 2, all *P* < 0.0001 Table 2). The upright position caused a more pronounced MAP fall in the cOH/SH+ group than in the cOH/SH− group (*P* = 0.0003, Fig. 2). In the entire cOH group, higher supine SBP was related to a larger SBP fall (*r* = −0.470, *P* = 2.4 × 10^−8^, Figure S1). The tilt-induced SBP response in controls did in contrast not depend on baseline SBP (*r* =  0.012, *P* = 0.863, Figure S1). When the SBP change after tilt was expressed as a difference between supine and fourth minute SBP, the fall in the high DBP group was higher (53 ± 30 mmHg) than in the low DBP group (33 ± 15 mmHg, *P* = 0.0004). However, the ratio of upright to supine SBP did not differ between those with high DBP (0.70 ± 0.15 mmHg) and those with low DBP (0.75 ± 0.10 mmHg, *P* = 0.09).

We will discuss differences between SH+ and SH− groups within patients and controls, with regards to the three BP determinants HR, SV and TPR.

#### Heart rate

HR did not differ between SH+ and SH− groups for patients or controls in either the baseline supine or the upright condition (Table 2 and Fig. 2).

#### Stroke volume

Supine SV did not differ between SH− and SH+ groups for cOH patients or controls (controls: *P* = 0.288, cOH: *P* = 0.151, Table 2). SV dropped immediately after tilt in all four groups. The fourth minute standing SV values did not differ between SH+ and SH− groups in controls (*P* = 0.498) nor in cOH patients (*P* = 0.673, Table 2).

#### Total peripheral resistance

Within the control group, we found a trend for higher supine TPR in the SH+ group than in the SH− group (control/SH− TPR=1.0, control/SH+ TPR=1.6, *P* = 0.0067, Table 2). In patients, the SH+ group showed a higher TPR in the baseline supine position than the SH− group (cOH/SH− TPR=1.0, cOH/SH+ TPR=1.4, *P* = 1.5 × 10^−5^, Table 2).

TPR showed large differences between cOH patients and controls within the SH+ and SH− groups (Fig. 2). When tilted to an upright position, TPR increased more and ended higher in the control/SH+ group than in the cOH/SH+ group, in whom TPR did not increase after head-up tilt (fourth minute: control/SH+ TPR = 2.1, cOH/SH+ TPR = 1.3, Fig. 2). Due to the different response to tilt of this cOH/SH+ group, the TPR in the fourth minute of tilt did not differ for the cOH/SH− and cOH/SH+ groups (cOH/SH− TPR=1.1, cOH/SH+ TPR = 1.3, *P* = 0.016; Table 2).

### Logratio analysis

**FIGURE 3:**
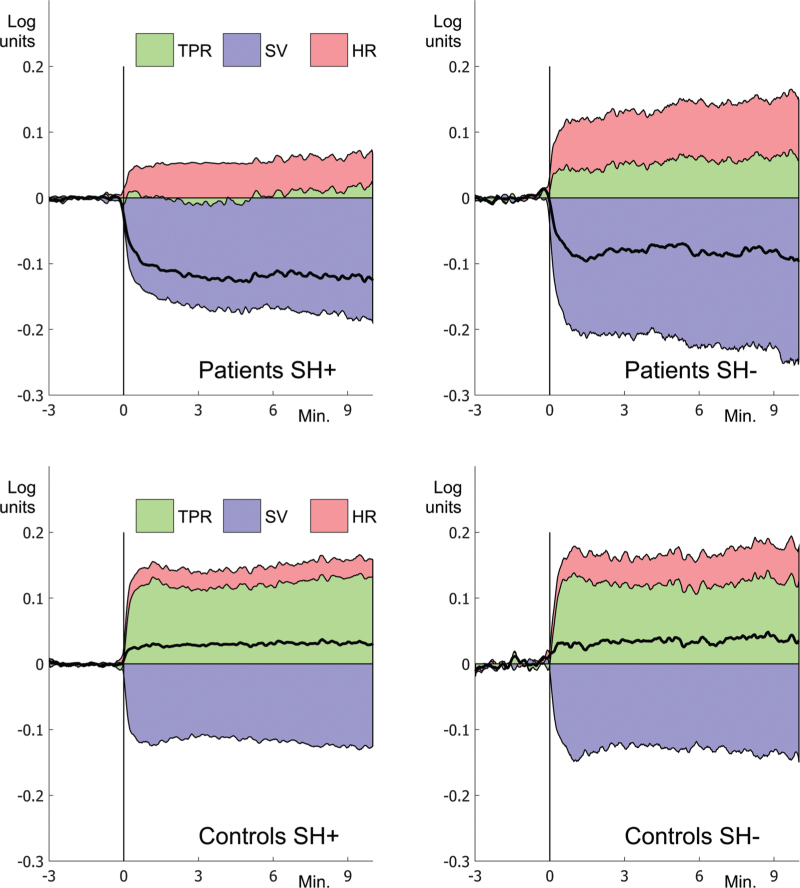
Logratio analysis of the head-up tilt. Data are shown for patients and controls, both split into those with (SH+) and without (SH−) supine hypertension. The vertical line at *T* = 0 min shows the moment of completion of head-up tilt. The respective influence of heart rate (HR; red), total peripheral resistance (TPR; green) and stroke volume (SV; purple) on mean arterial pressure (MAP; black line) are shown as cumulative areas. A positive logratio (LR) value signifies a positive effect on the MAP, whilst a negative LR value signifies a negative effect of the parameter on the MAP. The cumulative values of all three parameters at each point in time result in the MAP.

Figure 3 shows cumulative logratio values of HR, SV and TPR and their relation to MAP. Within the control group, MAP_LR_, HR_LR_, SV_LR_ and TPR_LR_ did not differ between SH+ and SH−, showing that the *relative* contribution to standing MAP in controls did not depend on SH (Fig. 3 and Table 2).

#### Mean arterial pressure

In the cOH group, MAP_LR_ did not differ between the SH+ and SH− groups (cOH/SH−: MAP_LR_ = 0.064 log units, cOH/SH+: MAP_LR_ = 0.090 log units, *P* = 0.047, Table 2 and Fig. 3). MAP_LR_ did not differ within the control group either (control/SH−: MAP_LR_ = 0.032 log units, control/SH+: MAP_LR_=0.027 log units, *P* = 0.643 (Table 2 and Fig. 3).

#### Heart rate

HR_LR_ was positive after tilt, indicating an increase and did not differ between the SH− and SH+ groups, neither within patients nor within controls (Table 2).

#### Stroke volume

SV_LR_ was negative, indicating a decrease; in cOH patients, SV_LR_ showed a larger SV decrease in the SH+ than in the SH− group (*P* = 0.00145, Table 2). In the control group, SV_LR_ did not differ between SH+ and SH− subgroups.

#### Total peripheral resistance

Within the control group, TPR_LR_ did not differ between SH+ and SH− (*P* = 0.709, Table 2 and Fig. 3). For patients, however, TPR_LR_ was positive in the cOH/SH− group and negative in the cOH/SH+ group. In other words, TPR decreased in the SH+ group but increased in the SH− group (*P* = 0.0025, Table 2 and Fig. 3a).

#### Interindividual variability

Pronounced individual variation in contributions of the three haemodynamic variables to MAP was apparent in the fourth minute logratio values (Figure S2). Within the cOH group, the most severe orthostatic hypertension occurred in patients with a negative TPR_LR_, that is, a decrease of TPR. This held for both SH+ and SH− patients. In the cOH/SH+ group, 27 (54%) individuals had a decrease of TPR, coinciding with large reductions in MAP regardless of initial supine BP. Within the cOH/SH− group, only six (20%) cases had a decrease of TPR, also mainly regarding those with larger reductions of MAP_LR_. The proportion of those with a reduction of TPR was larger in cOH/SH+ patients than in cOH/SH− patients (Fisher's exact test, *P* = 0.0045).

## DISCUSSION

We confirmed that high TPR was the dominant mechanism explaining high supine BP; in our study, this held both in those with and without cOH. Higher supine BP was accompanied by more severe cOH in cOH patients. We will show that this relation between severe SH and severe cOH is due to a seemingly paradoxical behaviour of TPR: while cOH patients with SH failed to increase TPR after tilt, they still displayed an excessively high TPR value in the supine position.

### Supine hypertension

We confirmed earlier findings stating that higher supine BP was linked to higher TPR in patients with pure autonomic failure [14]. In our results, this held true for cOH/SH+ patients and the hypertensive controls, with MAP and TPR at similar levels between those two groups. The main difference between cOH/SH+ and hypertensive controls was that TPR did not increase after tilt in cOH patients.

This failure to increase TPR further when needed is clearly visible in Fig. 2a and is consistent with previous literature [13,24,25]. Consequently, the upright TPR was not high enough to compensate for gravitational demands, resulting in more severe cOH in SH+ than in cOH/SH−. The importance of TPR to these group differences is clearly apparent in the logratio analysis in the form of a small TPR_LR_ for the cOH/SH+ group. To our knowledge, this study is the first to examine individual variation in the interplay of the different haemodynamic variables.

### Severity of orthostatic hypotension

We showed that supine BP, and thus SH, was related to the severity of cOH: higher supine BP correlated with a large BP reduction upon tilt. The close relation between DBP and SBP explains why results for DBP were very similar to those for SBP. Of interest, this held for absolute differences, but if the change of MAP after tilt was expressed as a ratio (e.g. logratio), the ratio did not differ between SH+ and SH− cOH patients (Table 2).

The finding that more severe cOH was linked to higher supine BP complicates treatment decisions, as severe SH requires antihypertensive measures and cOH antihypotensive ones [26].

The mechanisms underlying SH may well differ between neurogenic causes. In multiple system atrophy patients, SH has been ascribed to residual sympathetic tone, which may not apply to other disorders [15,27]. In Parkinson's disease, sympathetic denervation of vessels and the heart and concurrent hypersensitivity of cardiac beta-adrenoreceptors have been thoroughly documented, and hypothesized to be concurrent with parasympathetic dysfunction contributing to orthostatic hypotension [28,29]. Persons with orthostatic hypertension and sympathetic denervation had higher BP than those with orthostatic hypertension and intact sympathetic innervation, but no vascular resistance was reported [29].

We explored whether SH occurred more often in those with neurogenic causes of orthostatic hypertension; for this purpose, we first defined neurogenic orthostatic hypertension as cOH with Parkinson's disease, multiple system atrophy (MSA), pure autonomic failure (PAF), unspecified nOH or diabetes mellitus. Supine SBP did not differ between those with nOH (157 ± 30 mmHg) and without nOH (147 ± 35mmHg, *P* = 0.28). Proportions of SH+ and SH− did not differ either (chi-square, *P* = 0.31). We repeated the analysis counting only Parkinson's disease, MSA and PAF as nOH; this did not alter the results.

### Alternative mechanisms

The orthostatic BP fall in the cOH/SH+ group was clearly due to a failure of TPR to increase, which strongly suggests sympathetic damage in the sense of deficient vasoconstrictor ability. However, in these same patients, high supine BP was due to high TPR. This begs the question how patients unable to achieve sympathetic vasoconstriction in the upright position can have a high TPR in the supine position. In the cOH/SH− group, the TPR increase was also blunted, but less than in the cOH/SH+ group (Table 2). Undoubtedly, the baroreflex does not function properly in neurogenic cOH [30], and most of our patients had causes of neurogenic cOH [17].

We first reason that a complete paralysis of the baroreflex should incapacitate the ability to achieve high TPR through sympathetic vasoconstriction, both supine as well as upright. Secondly, we posit that autonomic function is most important when quick changes are needed, such as immediately after standing up. If these assumptions are true, then the high supine TPR in those with cOH/SH+ is only compatible with autonomic failure if another – slow acting – factor causes high TPR in this situation. Examples of such slow effects may be the stated residual sympathetic tone or hypersensitivity to circulating neuro-humoral factors [6,31]. In addition, endocrine humoral responses may also play a role. The well known BP overshoot after tilting back, regularly observed in those with autonomic failure [32], also suggests that slow-acting factors continue to maintain TPR and thus high BP after tilting back to the supine position.

Previous works have already hypothesized the influence of neurohumoral factors in this process [33–37]. We stress that TPR reflects vasoconstriction of any cause, not just vasoconstriction due to sympathetic nerve action. Circulating catecholamines have been studied most often, especially noradrenaline in Parkinson's disease, in which sympathetic denervation reduces plasma noradrenalin levels, and an artificial increase in noradrenalin causes BP to rise rapidly [33]. Angiotensin II has been found to be elevated in cOH patients with SH [34], which could also explain high TPR. Vasopressin is primarily influenced not only by the baroreflex but also via angiotensin II, and causes vasoconstriction. In more centrally caused baroreflex failure, the release of vasopressin is reduced. This is most often observed in those with more severe hypotension, who often rely on a vasopressin response [35–37]. Accordingly, vasopressin is an important BP regulator in autonomic failure [38]. These neurohumoral factors may explain the seemingly paradoxical behaviour of TPR we found here, perhaps in combination with other factors such as residual sympathetic tone. In short, TPR may rely on the expression of actions that work at different speeds: autonomic failure may preferentially impair fast mechanisms and its failure, therefore, becomes most notable when fast action is needed, for example, when standing up. In contrast, slow acting factors persist and cannot be countered quickly, explaining SH, at least in part.

### Limitations

The results underline the well known importance of TPR in BP regulation and fit well with a major autonomic contribution to all cases of cOH in the present article [17]. As such, the data fit with the proposition that all cOH may be neurogenic in nature, with a variable contribution of nonneurogenic factors [39]. We did not measure neurohumoral factors to assess whether they help explain the behaviour of TPR.

The control group did not reflect perfectly healthy individuals but formed an age-matched and sex-matched population, including people using various types of medication. Medication can influence the determinants of BP but are unlikely to explain differences between groups, as medication was used in both groups. More importantly, in an analysis of haemodynamic medication effects in the present study group, we found that the results did not change after exclusion of those using BP medication [17]. While the resulting groups are not pathophysiologically pure, they do represent patients as seen in daily practice.

Finally, the study is based on Modelflow data, meaning SV and TPR are estimated, not measured directly. We stress that there is no technique to measure TPR directly, and Modelflow is best at estimating relative alterations of BP components, which we did in the present study [22]. Modelflow may be incorrect when BP changes extremely quickly as during vasovagal syncope, but the current study focused on measures in the fourth minute after tilt and is therefore not subject to these shortcomings [19]. Relative changes of TPR and cardiac output (the product of HR and SV) can be reliably derived using Modelflow [40].

### Perspectives

A distinction between specific causes of cOH may yield interesting results when investigating the response of TPR and HR in different neurological and neurodegenerative disorders. Studies analysing differences and similarities are warranted to unveil the diverse underlying neurological and neurohumoral mechanisms. The large interindividual variability noticed here deserves further exploration; for instance, those with profound decreases of SV and those with profound TPR failure and SH may require different cOH treatments leading to personalized medicine.

## ACKNOWLEDGEMENTS

Funding: this research was funded in part by the Michael J. Fox Foundation for Parkinson's Research (MJFF grant 020200). For the purpose of open access, the author has applied for a CC BY public copyright license to all Author Accepted manuscripts arising from this submission.

Author contributions: J.G.v.D., I.A.R. and R.D.T. conceptualized the manuscript. A.H.S., B.S.G. and F.K. gathered and interpreted tilt test and clinical data. R.H.R., M.J.H. and J.G.v.D. conducted data analysis. A.H.S. and J.G.v.D. drafted the manuscript. All authors had access to the data, revised and approved the manuscript.

### Conflicts of interest

R.D.T. received speaker or consultancy fees from Theravance Biopharma, Arvelle, Eisai, Zogenix, Xenon, Angelini, UCB, and Novartis and research funding from NewLife Wearables.

## Supplementary Material

Supplemental Digital Content
