# Supplementary material for: Haemodynamic determinants of supine hypertension in patients with classical orthostatic hypotension
Source: J Hypertens. 2025 Nov 13;44(2):360–8. doi: 10.1097/HJH.0000000000004194 (PMC12746785; doi:10.1097/HJH.0000000000004194)
Supplement: Supplemental Digital Content [file jhype-44-360-s001.docx]

**Supplementary material**


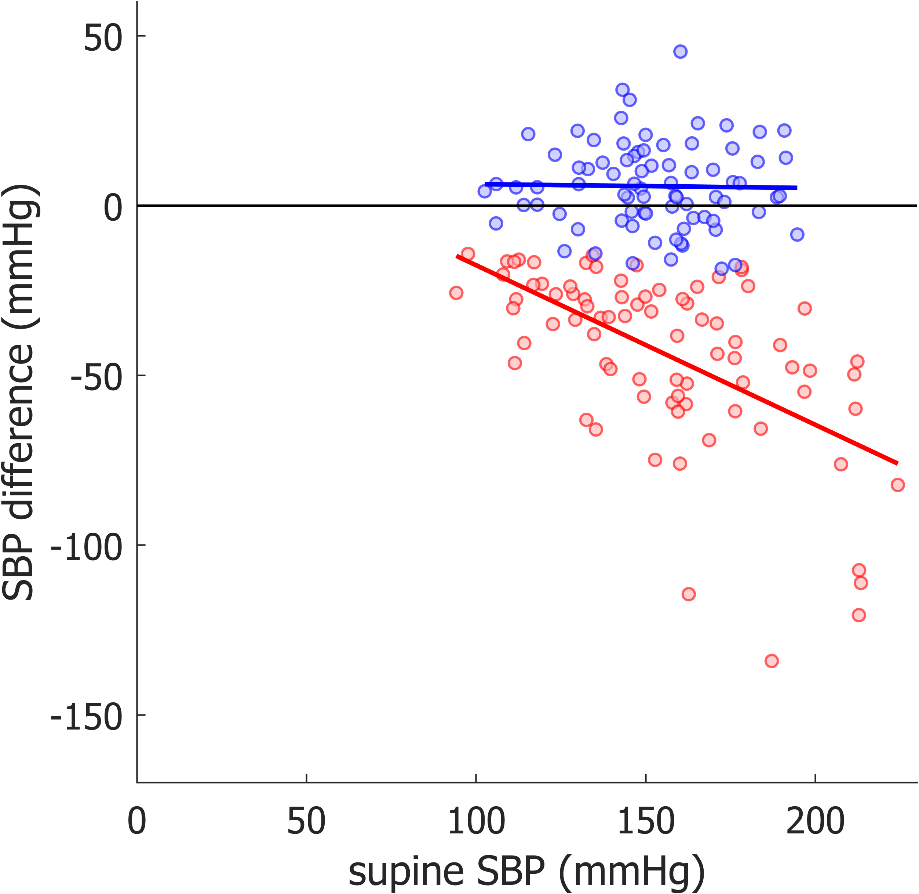


**Figure S1:** *Change in systolic blood pressure (SBP) after head-up tilt*.

The SBP difference is defined as the change of the supine SBP value to the fourth minute tilted value, meaning a negative SBP difference shows a reduction upon tilt. Blue dots and the blue line show control values (n = 80), and red dots and the red line show cOH data (n=80).


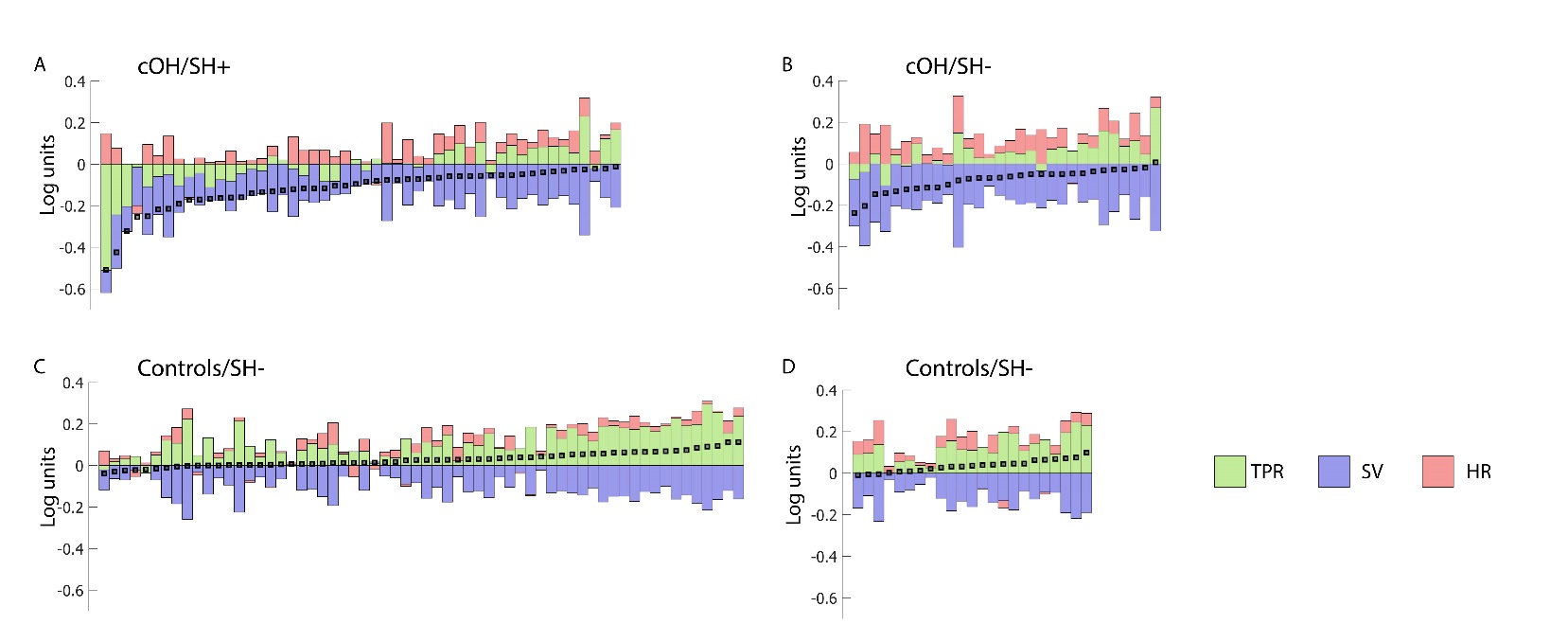


**Figure S2*:*** *Individual logratio analysis of the fourth minute after head-up tilt*.

The respective contributions of heart rate (HR; red), stroke volume (SV; purple) and total peripheral resistance (TPR; green) to the upright mean arterial blood pressure are shown for individual cases. Cases were divided by patients and controls groups as well as by presence of supine hypertension (SH+) or its absence (SH-). Within each group subjects were sorted based on the relative change of MAP in the fourth minute after tilt, with the largest reduction in MAP shown on the left. A) classic orthostatic hypotension (cOH) and SH+ group, B) cOH/SH- group, C) control/SH+ group, D) control/SH- group. In general, the increase in supine MAP from left to right within each group is paralleled by an increase in TPR.
